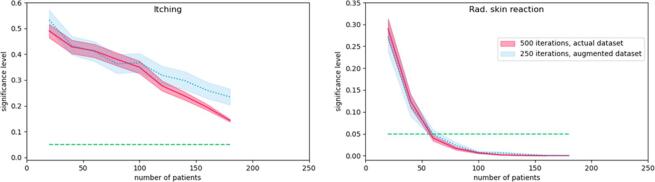# On the sensitivity of PROMs during breast radiotherapy

**DOI:** 10.1016/j.ctro.2022.100572

**Published:** 2022-12-26

**Authors:** Gerd Heilemann, Andreas Renner, Daniela Kauer-Dorner, Stefan Konrad, Inga-Malin Simek, Dietmar Georg, Joachim Widder

**Affiliations:** Department of Radiation Oncology, Comprehensive Cancer Center Vienna, Medical University Vienna, Währinger Gürtel 18-20, 1090 Vienna, Austria

**Keywords:** Patient reported outcome measures, Breast cancer, Quality of life, Partial breast, Irradiation, eHealth

## Abstract

**Purpose:**

To investigate the sensitivity of patient-reported outcome measures (PROMs) to detect treatment-related side effects in patients with breast cancer undergoing external beam photon radiotherapy.

**Methods:**

As part of daily clinical care, an in-house developed PROM tool was used to assess side effects in patients during a) whole-breast irradiation (WBI) to 40 Gy, b) WBI with a sequential boost of 10 Gy, and c) partial-breast irradiation (PBI) to 40 Gy.

**Results:**

414 patients participated in this prospective study between October 2020 and January 2022, with 128 patients (31 %) receiving WBI, 241 (58 %) receiving WBI followed by a sequential boost, and 50 patients (12 %) receiving PBI. Significant differences in the reported toxicities (itching, radiation skin reaction, skin darkening, and tenderness and swelling) were reported between the WBI cohorts with and without boost (p < 0.001, p < 0.001, p < 0.001, and p = 0.002, respectively). The comparison of PBI with WBI (no-boost) yielded significant differences for radiation skin reaction (p < 0.001).

**Conclusion:**

The results highlight the high sensitivity of PROMs to detect treatment-related side effects in patients with breast cancer. Thus, PROMs may be a valuable tool for quality control and may support evidence-based learning from real-world data originating from daily routine care.

## Highlights

• PROMs are very sensitive in detecting treatment induced side effects in radiotherapy of breast cancer.
• Sensitivity simulation allows to predict trends between two cohorts when analyzing patients in a real-world setting.
• Framework could be used for rapid learning to determine runtime of learning cycles in evidence generating learning healthcare system.
• Patients reported significantly more side effects after receiving a boost of 10 Gy.

## 1 Introduction

Overall oncological outcomes for patients with breast cancer are steadily improving [1]. Therefore, the management and prevention of side effects of treatment components and their impact on health-related quality of life (HRQL) have become increasingly important [2].

Over the past decade, patient-reported outcome measures (PROMs) have attracted growing attention, even in routine clinical care. Randomized prospective data demonstrated a favorable effect on HRQL compared to routine physician-centered symptom monitoring when PROMs were systematically included in clinical care [3]. PROMs have shown merit in assessing acute symptomatic toxicities in radiation oncology [4]. Moreover, patients' compliance to participate in PROM programs and provide valuable and actionable data could significantly be improved by moving the surveys and data collection to digital platforms [5]. The accessibility of these new possibilities may significantly reduce the barriers for patients to participate and thereby improve compliance, reducing data incompleteness. This might allow efficient monitoring of the direct effects of radiation therapy in almost real-time based on real-world data retrieved from consecutive non-selected patients.

Administering a boost dose in whole-breast irradiation (WBI) therapy has been shown to improve outcomes in selected indications. Still, it is known to lead to a small but significant increase in late fibrosis [6]. Little is known about the acute patient-rated experience of receiving a booster dose, especially when contemporary hypofractionated treatment is administered (15 fractions to 40 Gy), which leads to less overall skin toxicity compared with conventional fractionation (25 fractions to 50 Gy) [7]. Moreover, partial-breast irradiation (PBI) provides an alternative to WBI for some patients, further reducing the overall exposure to radiation and consequentially decreasing radiation-related toxicities [8]. Ultrahypofractionation in breast radiotherapy is presently being introduced at many institutions, and accompanying toxicity surveillance agrees with current recommendations [7].

Recently, our clinic implemented an in-house-developed PROM tool to monitor the clinical impact of new forms of treatments in real-time. The purpose of this study was twofold: a) to analyze the sensitivity of PROMs as a tool to monitor and detect treatment-related acute toxicities in a real-world setting and b) to provide a use-case to test potential differences between patient-reported toxicities in different cohorts (i.e., boost vs no-boost vs PBI) undergoing breast radiotherapy, where typically physician-rated toxicity is very low. Using real-time monitoring of patients with breast cancer, we aimed to explore whether active PROM surveillance might be an early indicator for differences in even low-grade toxicities experienced by patients.

## 2 Methods

The Patient Experience Data in Radiation Oncology (PEDRO) study was approved by the Institutional Review Board of the Medical University of Vienna (EK 2184/2019) and was registered under the ClinicalTrials.gov NCT05224297.

### Patients and inclusion criteria

All patients treated for breast cancer between October 2020 and January 2022 (16 months) were eligible after signing informed consent to participate in the study. Patients received hypofractionated WBI or PBI to 40 Gy in 15 fractions. WBI was followed by a risk-adapted sequential boost of 10 Gy in four fractions if indicated. All treatment plans were based on CT imaging, and left-sided treatments were planned and delivered in deep-inspiration breath-hold with a surface-guided treatment technique. The typical treatment plan consisted of tangential photon beams. In rare cases, volumetric modulated arc therapy (VMAT) was used when treating physicians felt the need to improve dose distributions in the entire breast to fulfill normal tissue constraints.

### Outcome measures and surveys

Acute toxicity was scored using PRO-questionnaires. Consenting participants answered pre-defined questions retrieved from the PRO-CTCAE catalog [9]. The questionnaires are listed in the appendix. The first PRO data was assessed before the start of the treatment on the day of the first irradiation. This time point was defined to be the baseline. Then, surveys were carried out weekly on tablets in the outpatient clinic or online in our web-based application for the duration of the treatment, with the last data point on the day of the last fraction (±max one day).

The PROM application, the app server and the database infrastructure were all developed in-house, and the instrument is directly linked to the patient chart in the oncology information system (MOSAIQ, ELEKTA, Sweden).

### Statistical analysis

The general study population was described using descriptive statistics. The boost vs no-boost groups were analyzed for significant differences with respect to four distinct endpoints: itching; radiation skin reaction; skin darkening; breast swelling and tenderness. Three different time points were used to test the hypothesis that there is no difference between patients with and without a boost: a) Baseline, b) after 40 Gy, and c) after 50 Gy for patients receiving a boost. The two groups were compared using the Mann-Whitney *U* test with the significance level set to 0.05.

Additionally, the impact of sample size on the differences in reported radiation induced toxicities between the two groups was analyzed. This was done with a bootstrapping method, simulating the runtime of the study with a growing study cohort size by randomly picking from all subjects. Two approaches were designed: 1) bootstrapping from the actual study data set and 2) bootstrapping from an artificially augmented data set to analyze the generalizability of the sensitivity analysis on a broader population. The augmented data set was generated by triplicating the study data set.

All data processing and statistical analysis was done in Python using the SciPy library.

## 3 Results

### Patient cohort

A total of 419 patients with breast cancer participated in this real-world PROM setting, comprising more than half of all patients with breast cancer treated at our institution in this period. Non-participation was almost exclusively due to organizational reasons and the failure of physicians to invite patients for inclusion. Patient refusal to participate after information was well below 2 %. 241 patients (57.5 %) were treated with a sequential boost, 128 patients (30.5 %) received no boost, and 50 patients (11.9 %) were treated with PBI (see Table 1). The median age was 59 and 64 years, respectively.

**Table 1:** Descriptive statistics of the study cohort.

	All patients (n = 419)	With boost (n = 241)	No boost (n = 128)	Partial-breast (n = 50)	p-value (Kruskal-Wallis)
*Age*					3.56e-10
Median (IQR)	61 (53–70)	64 (51–66)	59 (52–73)	71 (62–80)	
*T-Staging*					0.578
0	3	2	1	0	
1	236	133	71	32	
2	85	61	18	6	
3	10	6	4	0	
4	9	4	5	0	
X	54	26	20	8	
pTis	22	9	9	4	
*N-Staging*					0.018
0	281	160	87	34	
1	63	47	14	2	
2	4	3	1	0	
3	2	0	2	0	
+	2	2	0	0	
x	67	29	24	14	
*M−Staging*					0.124
0	416	241	126	49	
1	3	0	2	1	
*ER*					1.04e-05
positive	344	183	113	48	
negative	67	56	9	2	
unknown	8	2	6	0	
ER					
*PR*					0.001
positive	300	158	98	44	
negative	111	81	24	6	
unknown	8	2	6	0	
*Her2*					0.006
positive	75	51	23	1	
negative	335	188	99	48	
unknown	9	2	6	1	
*Technique*					0.148
VMAT	38	25	14	10	
3DCRT	381	216	114	40	

### Boost vs no-boost

**Fig. 1:**
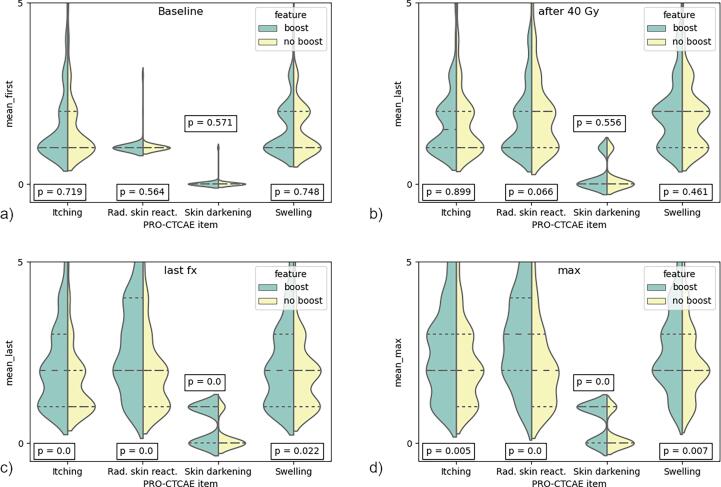
Violin plots for the different endpoints at 3 different time points (baseline, after 40 Gy, and at last fraction) for WBI (boost vs no-boost). Additionally, the maximum reported toxicity for each end point was compared between the two groups (max). The values indicate the score from 1 – none up to 5 – very severe (itching, radiation skin reaction and swelling) or 0 – no and 1 – yes (skin darkening).

At baseline, patients in both groups reported low toxicities for the endpoints (Fig. 1a) with no differences between the two groups for itching (p = 0.72), radiation skin reaction (p = 0.56), skin darkening (p = 0.57) and tenderness and swelling (p = 0.75). After 40 Gy, the reported side effects were higher than at baseline for both groups, without any significant differences between the groups: p = 0.90, p = 0.07, p = 0.56, and p = 0.46 for the four endpoints, respectively (Fig. 1b).

However, after receiving the boost of 10 Gy, patients in that group reported significantly higher toxicities (Fig. 1c): itching (p≪0.001), radiation skin reaction (p≪0.001), skin darkening (p≪0.001) and tenderness and swelling (p = 0.02). Similarly, a comparison of the overall maximum reported side effects (Fig. 1d) resulted in significant differences between both groups for all four investigated side effects, with p = 0.01 (itching), p ≪ 0.001 (radiation skin reaction), p ≪ 0.001 (skin darkening), p = 0.01 (tenderness and swelling).

**Fig. 2:**
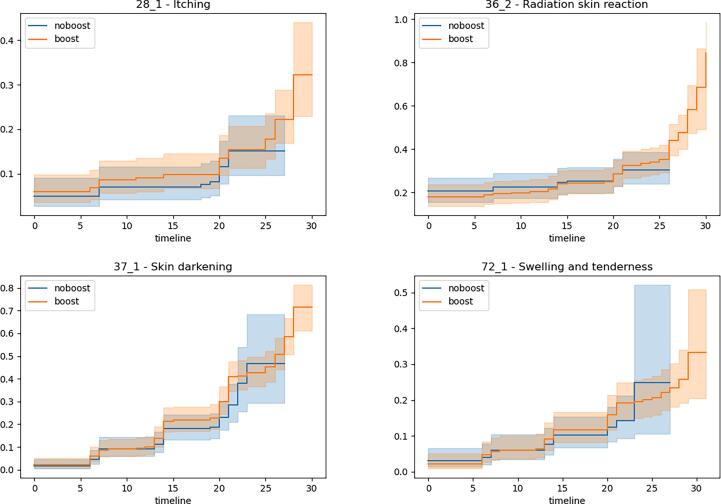
Cumulative risk curve of the boost and no-boost cohort for the different end points. An event was defined to be a score of 4 or 5 for itching, radiation skin reaction and swelling/tenderness and a 1 in case of the binary endpoint skin darkening.

The cumulative risk of developing “severe” toxicities is plotted in Fig. 2. A severe event was defined as a reported score of 4 or higher (i.e., severe and very severe) for the endpoints itching, radiation skin reaction, tenderness/swelling, and a true statement in the binary endpoint of skin darkening. Severe toxicity (G3-4) was rare and did not significantly differ between treatment schedules.

## Sensitivity analysis of PROMs

**Fig. 3:**
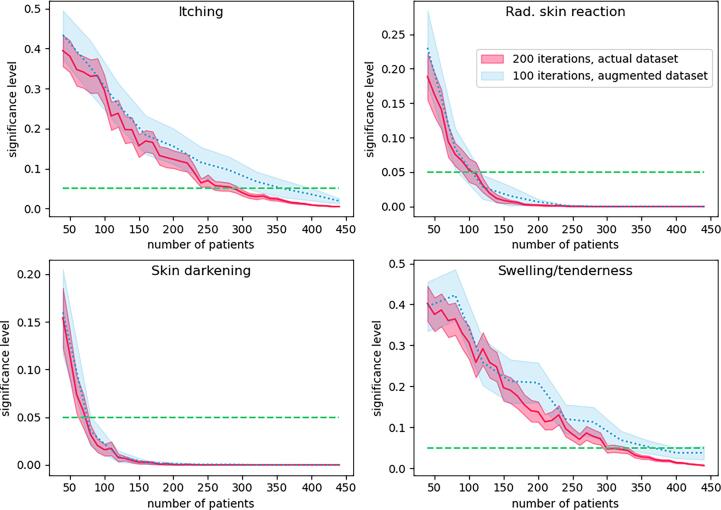
Bootstrapping to simulate the sample size effect on the significance level between boost and no-boost. The graphs show the different simulations with the actual and an augmented dataset (3 × actual dataset).

The sensitivity of PROMs to detect differences between the two groups (boost vs no-boost) was not the same for the four endpoints (see Fig. 3). Two simulations were performed to determine how the dichotomy between the two groups stabilized for a growing number of subjects in the study using 1) 200 random picks of patients in the actual study cohort and 2) 100 random picks of patients from an augmented study cohort data set.

Repeatedly sampling the data set with random replacement in the actual study cohort yielded robust significance levels at around 300 patients for itching, 120 patients for radiation skin reaction, 80 for skin darkening, and 340 for swelling and tenderness. The same simulation using the augmented data set required a slightly higher number of patients to achieve robust significance levels.

### Partial-breast irradiation and early-stage detection of differences in cohorts

With 50 patients, the PBI cohort was relatively small compared to the (no-boost) WBI group. A graphical analysis can be found in the supplementary data (Suppl. data 1). Significant differences were reported for radiation skin reaction (p < 0.001). The other items showed no difference. However, the simulation of increasing sample size clearly indicates a trend of continuously decreasing p-values.

## 4 Discussion

Using PROMs in a real-world setting to monitor acute effects made it possible to pick up differences between patients with breast cancer receiving a boost vs no-boost. In some cases (e.g., “radiation skin reaction” and “skin darkening”), this already happened with valuable patient feedback after a relatively short study runtime of around 100 patients. Other side effects required larger sample sizes (i.e. 300 and more for the endpoints “itching”, “tenderness and swelling”). This highlights the high sensitivity of PROMs in a real-world setting.

The comparison of PBI vs (no-boost) WBI, which was based on a significantly smaller sample size, yielded significant differences for “radiation skin reaction” only. For “itching,” the p-values for the analysis of PBI vs WBI showed a trend toward the significance threshold (α = 0.05). Again, this underlines the surprising sensitivity of PROMs, suggesting the instrument as a method for early analysis of low-grade toxicity trends even in small patient sample sizes. Indeed, acute toxicities in PBI are reported less frequently than for WBI, as demonstrated in larger study cohorts [10].

In their systematic review from 2021, Price *et al.* [11] outline the methodological and analytical approaches necessary for establishing learning healthcare systems (LHS) in oncology. These mostly conceptual frameworks promise the swift translation of clinical evidence generated from real-world data into clinical practice. However, while Price *et al.* demonstrate more than a dozen applications of “rapid learning” concepts in oncology, they point out that none of the studies were driven by the clinical outcome, in contrast to the present study.

Our in-house developed PROM tool aimed to provide a framework that allows for real-time monitoring of clinical outcomes (i.e., toxicities) at a large scale. The results of this study allowed us to estimate the sensitivity of the PROM tool. In the process, we developed a simulation method that can help to estimate the runtime of plan-do-study-act cycles within an LHS framework.

Active surveillance of PROMs and simulating the runtime of a monitoring phase (e.g., after introducing a new treatment option) allowed to detect trends in reported side effects between groups receiving different radiation treatments. The bootstrapping methods showed that the results are reproducible, even for a larger population, thus demonstrating their generalizability.

A key issue incorporating PROMs to monitor the impact of changes in radiation treatment concepts and techniques in terms of even subtle toxicities is to – at the same time – reduce barriers on a patient level, on the health provider level, and on a technical level [12]. This is crucially important because the sensitivity of the PROM tool will decrease if only a fraction of all patients is included. In the PEDRO study, we addressed all three dimensions mentioned above. We offered our patients the options of a) on-site participation on stationary tablets (with optional assistance by our study assistants) or – at a later stage – b) completing the surveys on their smartphone/computer via a web-based application, where patients will receive the questionnaires to their personal electronic devices. We believe that this improved accessibility of our hybrid approach, e.g., by allowing patients to decide when and where they participate at their own leisure and comfort, has significantly contributed to the high level of participation and compliance with only a few study dropouts. During the implementation phase, recruitment and participation levels even continued to increase, suggesting growing levels of confidence both for patients and professionals in using PROMs as part of daily clinical care. Another important aspect was the high level of automation of our software tool, which triggers responses to questionnaires by the treatment schedule in the oncology information system.

In the future, this in-house developed tool will be used to assess PROMs at follow-up moments, e.g., at 3, 6, 12, 24, and 36 months – or any other appropriate point in time – after radiotherapy. Recruitment for this stage is ongoing, and the first surveys have been conducted as we aim to analyze differences in long-term side effects between treatment regimens and treatment techniques in the long run. Furthermore, the data from this study will be the basis for monitoring the implementation of highly accelerated treatment protocols (ultrahypofractionation) in the treatment of breast cancer at our institution (e.g., FAST Forward [13]).

This report describes important methodological cornerstones for implementing PRO-based monitoring of differences between and developments of novel treatment regimens and techniques. An obvious limitation of the study is the absence of an assessment at one week after the last treatment fraction in the non-boost cohort. Due to this limitation, it cannot be excluded with certainty that patients not receiving a boost dose might experience the same increase in side effects one week after the last fraction as those receiving four extra doses. Such an additional assessment one week post treatment would have been infeasible for a real-world setting. This question will be addressed when the boost dose is integrated simultaneously into the treatment in the near future. The technique (3DCRT or VMAT) was not found to be relevant for developing side effects. Patients in this study were primarily treated with 3DCRT (>90 %), but the primary technique in the future will be VMAT. The impact of this transition will be monitored with our tool but is out of this study's scope.

However, the present report is primarily intended methodologically: to demonstrate the sensitivity of a table-based PRO tool as an unexpectedly sensitive instrument to measure even subtle treatment-induced side effects. The results rendered the instrument's utility for near-time quality control and improvement of radiation-treatment delivery and planning highly promising, and it will be further implemented for other indications and patient cohorts in a wide range of treatment settings.

## Declaration of Competing Interest

The authors declare that they have no known competing financial interests or personal relationships that could have appeared to influence the work reported in this paper.

## Figures and Tables

**Supplementary figure 1:**